# Validation and Assessment of Osteoporosis Self-Efficacy Among Iraqi General Population

**DOI:** 10.2174/1874434601812010076

**Published:** 2018-05-31

**Authors:** Mohanad Naji Sahib

**Affiliations:** Faculty of Pharmacy, Al-Rafidain University College, Palestine street, 10052, Baghdad, Iraq

**Keywords:** Arabic, General population, Osteoporosis, Preventive behavior, Self-efficacy, Calcium, Vitamin D

## Abstract

**Background:**

Poor quality of life, fractures and disability are the consequences of preventable osteoporosis.

**Objectives:**

The aims of this study were to validate and assess Osteoporosis Self-efficacy Scale (OSES-A) Arabic version among Iraqi general population.

**Methods:**

A cross-sectional study with a random cluster sampling method from the community was used. Forward–backward-forward translation method was used to translate the questionnaire from English to Arabic. Beside OSES-A, Osteoporosis Knowledge Tool (OKT) and Osteoporosis Health Belief Scale (OHBS) Arabic versions were used to assess osteoporosis preventive behaviours.

**Results:**

The results showed good face validity and reliability. The construct validity showed two factors which explain 80.86% of the variance. In addition, the result showed low self-efficacy score (658.43±222.014) with 83.33% were found to have low OSES-A level. There were significant associations between age, gender, and self-reported osteoporosis with OSES-A levels. In addition, there were significant differences between age, gender, marital status, family history of osteoporosis, self-reported osteoporosis and osteoporosis diagnosis or screening in relation to total OSES-A scores. Moreover, there were positive correlations between the OSES-A total score with total knowledge and health belief. Multivariate analysis revealed that OKT levels, OHBS levels, age and gender were predictors for OSES-A levels.

**Conclusion:**

This study showed good cultural adaptation and psychometric properties of OSES-A tool and could be used in any osteoprotective educational program.

## 1 INTRODUCTION

Osteoporosis (OP) is the main cause of hip, spine, and wrist fractures which is due to the decrease in bone mineral density [1]. Poor quality of life, costly rehabilitation, disability and premature death are the consequences of this preventable disease [2]. Both genders are at risk of OP [3]. As with other chronic diseases, prevention is critically important in preventing OP. Lifestyle modification is the key component in this prevention process to increase bone mass density. Managing modifiable risk factors like regular weight-bearing exercise, increasing calcium and vitamin D intake, reducing smoking and alcohol intake are the cornerstone in preventing OP [4-6].

Although knowledge is crucial for healthy life style behaviors, it is solely not enough for the changing behaviors [3, 7-9]. Healthcare professionals can develop and implement a specific educational program according to good understanding of populations’ belief and self-efficacy as changing lifestyle and healthy behaviors at a younger age will have a greater impact on the prevalence of OP as with other chronic diseases [10-13].

By definition, self-efficacy is the confidence of the person in terms of his/her capacity of coping with the difficulties in organizing and implementing healthy behavior activities [14]. Moreover, it is known that increasing the person’s self-efficacy perception is an effective way to gain positive healthy behaviors [15-18]. Therefore, increasing knowledge, health belief and strengthening the self-efficacy perceptions of the society are substantial to prevent osteoporosis [8]. Therefore, the aims of this study were to validate and assess the self-efficacy toward OP in Iraqi general population.

## 2 MATERIALS AND METHODS

### 2.1 Study Design and Participants

A community based, cross-sectional study was conducted from November 2016 to February 2017 in Baghdad city, Iraq. Baghdad city has two large areas named Al-Kharkh and Al-Rusafa. Three districts were selected by random cluster sampling method from these areas. The community pharmacies where the undergraduate students underwent training in these districts were used to invite the participants. Systematic samples were randomly selected. A structured interview included collection of the socio-demographic and translated OSES-A data. Each participant was interviewed individually by the researcher or a trained 5^th^ year undergraduate student after obtaining written or verbal informed consent. Some of participants gave only verbal informed consent because they considered it impolite behavior given that they had already given verbal consent for participation. The study protocol and ethical approval (including verbal informed consent) were approved by the Scientific Committee of Al-Rafidain University Collage, Baghdad, Iraq.

### 2.2 Sample Size

A recommendation suggested that at least 5 subjects per item are needed to evaluate the reliability and validity of a questionnaire [19]. The original OSES consist of 12 questions; therefore 60 participants were needed for the purpose of validation. However, for factor analysis, it is preferable to use 300 subjects [20]. Moreover, with this number of participants, it would be possible to discriminate between high and low correlations in measuring correlations [21]. Only 400 participants were accepted to be involved in this study, however, 45 of them were ineligible due to incomplete responses. Therefore, only 355 participants were selected for this study. Thirty participants of them were randomly selected for the pilot study in face validation step of the translated questionnaire and not included in the final analysis. Moreover, 25 participants from the sample population were randomly selected for test- retest within 1–2 weeks.

### 2.3 Instruments and Measurements

All participants completed the structured questionnaires including OSES-A. The original OSES is in English language and designed to assess self-efficacy of behaviors toward OP [22, 23]. The OSES is a twelve items rated by individual on a 100 mm visual analogue scale to assess the confidence in performing osteoporosis preventive behaviors. The OSES has two subscales namely: OSES-Exercise and OSES-Calcium. The possible total score range from 0 to 1200 with each subscale range score from 0 to 600. A cut-off point (858) was used to categorize the osteoporosis self-efficacy scores into two levels: low and high OSES-A levels [24].

Beside OSES-A, unpublished but valid and reliable Osteoporosis Knowledge Tool (OKT-A) and Osteoporosis health belief Scale (OHBS-A) Arabic version tools, were administered before OSES-A, respectively, according to the developer instructions. Also, the original OKT and OHBS are in English language and designed to assess OP knowledge and health beliefs about developing osteoporosis, respectively [23, 25]. The OKT is 24 multiple-choice items regarding risk factors and its prevention. From 0 to 24 is the possible score range and the highest value indicate the highest level knowledge score. While, the OHBS consist of 42- likert type scale (1 = strongly disagree, 5 = strongly agree). The total possible score range from 42 to 210. Cut-off points (14 and 169) were used to categorize OKT-A and OHBS-A scores into two levels: low and high, respectively [26, 27].

### 2.4 Instrument Translation and Face Validity

Forward–backward-forward translation method was used to translate the questionnaire from English into Arabic according to translation international guidelines including forward translation, reconciliation, reverse translation, debriefing [28-30]. The translation process was conducted by two independent, expert translators. Thereafter, an expert panel of eight clinical pharmacists and the researcher reviewed the Arabic version for reconciliation. Then, back-translation of the reconciled version was carried out by another two independent expert translators. Subsequently, discussions between the expert panel, translators, and the researcher were held to resolve any inconsistencies and check the capacity of the items to measure the construct that it proposes to measure then a final version was decided (face validity process) [31].

Finally, a pilot study was conducted by distributing the questionnaire to 30 participants and the questionnaire was modified according to their feedback after discussions between the expert panel, and the researcher. Those 30 participants were excluded from the final study outcome and analysis.

### 2.5 Construct Validity

Exploratory Factor Analysis (EFA) was examined to found the factor structures of OSES-A. A principal axis factoring method for extraction with direct Oblimin (Oblique) rotations was used for EFA. The criteria for EFA were: factor loading greater than 0.40, Kaiser–Meyer–Olkin (KMO) value (> 0.5), Bartlett’s test of sphericity (significant level < 0.05). The number of factors retain were depend on: Kaiser’s criterion (eigenvalue ≥ 1.0) and theoretical meaning of the rotated factors [32].

### 2.6 Reliability

Reliability with a minimum acceptable criterion above 0.5 was applied to measure the consistency of a measurement item [33]. The internal consistency was evaluated using Cronbach’s alpha and corrected item total correlations between the scales and their corresponding items (correlation of < 0.20 is considered poor). Pearson’s correlation coefficient was used to evaluate test–retest reliability [19].

### 2.7 Statistical Analysis

The statistical analysis of the validation processes included assessing construct validity and reliability (Cronbach’s alpha and test-retest). Descriptive statistics, percentages, and frequencies were used as appropriate. The chi square (χ^2^) test was employed for categorical variables to find any association, whereas for continuous data, Mann–Whitney U and Kruskal–Wallis tests were used to evaluate the differences between the groups when required. In addition, logistic regression analysis using backward method was used to identify the factors affecting OSES-A. Predictive Analytics Software (PASW) version 19.0 was used to analyze data in this study and significance level was set at *P* value <0.05.

## 3 RESULTS

### 3.1 Socio-Demographic

The age of the participants was a range between 18 to 87 years with an average of 41.82±12.452 years. Nearly 46% of respondents were male. About 79% of the respondents had educational level more than 12 years. About 23% of the respondents were single and around 38% had monthly income less than 500,000 Iraq Dinar (IQD; 1 US dollar is equivalent to 1,250 IQD). By employing the recommended scoring method, the mean scores (M±SD) of the OSES-A was 658.43±222.014 which considered low.

Table **1** shows the distribution of the two levels of osteoporosis self-efficacy and the demographic data results. Only 16.67% of the study population was found to have high OSES-A level. The results showed significant differences between the following independent variables in relation to total OSES-A scores: age, gender, marital status, family history of osteoporosis, self-reported osteoporosis and osteoporosis diagnosis or screening. In addition, there were significant associations between ages, gender, and self-reported osteoporosis with OSES-A levels (Table **1**). Furthermore, the results revealed low self-efficacy in all dimensions (less than 60%) with the lowest value appeared in the exercise subscales (51.03%).

### 3.2 Validity

#### 3.2.1 Face Validity

As a result of the extensive translation method and pilot testing, qualitative face validity was guaranteed.

#### 3.2.2 Exploratory Factor Analysis (EFA)

In this study, a principal axis factoring analysis method was conducted on the 12 items with direct Oblimin (Oblique) rotations. Upon examination of the correlation matrices, a majority of the results showed a correlation larger than 0.3. The Kaiser-Meyer-Olkin (KMO) value was 0.913 which indicated that the data set was appropriate for EFA as it was greater than 0.5 [32]. The last measure was the Bartlett’s Test of Sphericity which was found to be highly significant (χ^2^_(66)_ = 3835.496; *P*<0.001).

These results allowed us to identify a factor model using the EFA approach [34, 35]. In addition, the analysis revealed two factors, with eigenvalues greater than one that explained 80.86% of the variance, as shown in Table **2**.

In addition, the eigenvalues of Factor 1 (OSES-M Exercise subscale) and Factor 2 (OSES-M Calcium subscale) explained 55.51% and 25.35% of the variance, respectively. The entire results showed adequacy for factor analysis and with two domain (subscales) variables.

#### 3.2.3 Reliability

For the reliability, the Cronbach's alpha test of internal consistency for total OSES-A, 0.927 and it’s within the recommended acceptable result for reliability [33]. Test-retest reliabilities of OSES-A demonstrated significantly positive relationships in a sample of 25 subjects (*r*= 0.859, *P*<0.001). An initial Cronbach’s alpha result for the OSES-A test-retest group was 0.719, and after 1 to 2 weeks it was 0.798. These results demonstrated that OSES-A was reliable and stable. The corrected item-total correlation values, which is the reliability index, ranged from 0.556 to 0.795 (Table **3**). All items appeared to be suitable for retention depending on the meaningfulness of the items [19].

### 3.3 Multivariate and Correlation Analysis

Correlations were performed to determine the relationship between total OKT-A and OHBS-A with total OSES-A. There were positive correlations between total OSES-A score with total OKT-A score, *r*=0.274, and OHBS-A, *r*=0.238 (all *Ps*<0.01). The binary logistic regression revealed that OKT-A (categorical) and OHBS-A (categorical), age (continuous) and gender were predictors for total OSES-A (Table **4**) and the model explain about 84.70% of the dependent variable.

## 4 DISCUSSION

Self-efficacy is the opinion and the attitude to engage and maintain activity in face of obstacles [36]. To reduce the risk of future bone fractures, osteoporosis prevention by the mean of educational program is the most effective way [37, 38]. Moreover, before any educational program to be implemented, the knowledge, health belief and self-efficacy must be assessed so that the program could be tailored according to the required need for the population.

In this study, the OSES-A was carefully reviewed and revised by a panel of eight experts in the pharmacy field after forward–backward-forward translation for face validity process. The EFA of the OSES-A has a stable factor structure with two factors accounted for 80.86% of the variance, which was higher than other studies [24, 39, 22]. This result may be due to different culture setting *i.e.*, nations, cultures and times as compared with the US population where the original tool developed in 1991. This highlights the important of cross-cultural adaptation process even for well-established questionnaires.

The reliability of the OSES-A which is the consistency of a measurement item, was an excellent with overall Cronbach’s alpha (0.927). This value was comparable to the original OSES, Malaysian and Persian studies [24, 39, 22]. The test–retest reliability Cronbach’s alpha value after 1–2 weeks was higher than the initial value indicating that the respondents may be more aware and more confident in engaging in healthier behaviors. Therefore, this could be used in longitudinal studies to measure the change in self-efficacy level and, consequently, improve their outcome. The validity and reliability results revealed successful cultural adaptation.

The results showed low frequencies in all dimensions with low overall self-efficacy score. The total OSES-A score for men was significantly higher than women. In addition, after controlling gender, men in both age groups showed insignificant results, while for women, younger age (<45) had higher self-efficacy. The OSES-A exercise subscale for men (frequency 58.24%) was higher than women (frequency 44.89%), however, this result was insignificant. This is an important result as it showed that any educational program should be highly focuses on highly risk group (*i.e.*, women). Previous literatures showed that the self-efficacy scores for men were higher than women [40, 41]. Moreover, Nayak *et al.* showed that the health belief was higher in younger than older age groups which affect the final preventive behaviors [42]. However, any program should be taken in to account both age groups and genders. The above result was consistent with the significant correlations between OSES-A score and the OKT-A and OHBS-A. The results were consistent with other studies which showed that increasing knowledge and health belief were positively affect self-efficacy [41, 43, 44].

Furthermore, by controlling OKT-A, the respondents with a history of OP were negatively correlated with the total OSES-A scores and OSES-A exercise subscale only. This was obvious as the pain gained from OP would prevent those participants to engage in different exercise behaviors. Shin *et al.* showed that the commitment to engage any exercise were differed according to the severity of pain and the intervention must be tailored according to the subjects need [18]. The results showed low OSES-A exercise subscale score. Therefore, the educational program should be focused on that regular exercise not only increase bone strength, but improve mood and physiological function, reduce frequency of disease, increase the quality of life [45-49].

In Iraq, there are no free public gym centers, running in the streets is not acceptable culturally, and women engaged in exercise also not acceptable. Other study showed that inconvenience, cost and time were the factors barriers for engaging exercise [50]. To overcome these barriers and for better health, a new policies must be implemented like the availability of free of charge public gyms. Moreover, encouraging and changing the attitude and belief of the community regarding the engagement in the exercise (especially for women) should be enhanced.

The results showed low mean score for OSES-A calcium subscale. This result must be highlighted in any interventional program. Enhance the awareness of the general population should be focused on that calcium-rich foods not only improve bone health but also improve weight lost, decrease the incidence of metabolic syndrome, decrease blood pressure [51-55]. The educator should also emphasis on increasing the knowledge of the participants about what the alternatives which are not costly and suitable for their needs. Also, the participants should know that there is no association between dairy products and the metabolic disorders [55-57]. Besides that, adequate calcium intake in the adolescents age were the peak bone density developed must be highlighted too [58]. Therefore, the attitude of the respondents in this study could be increased by means of the initiation of an effective prevention program, increasing the risk reducing behaviors and good coping strategies.

This study cannot be generalized for all population as it is a cross sectional study. Nevertheless, the comprehensive translation and validation steps with a good sample size and cluster sampling method give a high impact for this study.

## CONCLUSION

This study showed good psychometric properties of OSES-A tool and could be used in clinical setting or with general population. Furthermore, increasing osteoporosis self-efficacy of Iraqi population in all dimensions (exercise and calcium intake) is warranted as changing lifestyle and healthy behaviors will have a greater impact on the prevalence of osteoporosis.

## Figures and Tables

**Table 1:** Demographic characteristics of participants; Data expressed as M±SD or frequency (percentage, %).

**Characteristics**	**Total samples (N=300)**	**Low self-efficacy** **(N=250)**	**High self-efficacy (N=50)**
12 item OSES-A score	658.43±222.014	591.96±174.429	990.80±104.371
OSES-A exercise	306.17±153.752	265.72±131.658	508.40±78.020
OSES-A calcium	352.27±112.667	326.24±99.098	482.40±82.575
**Age** ^a^ *	-	-	-
≤44	59.7	56.8	74.0
≥45	40.3	43.2	26.0
**Gender** ^b^ *	-	-	-
Male	46	43.2	60.0
Female	54	56.8	40.0
**Marital status** ^c^	-	-	-
Single	23	21.6	30.0
Not single	77	78.4	70.0
**Educational levels**	-	-	-
< 12 years	21	22.0	16.0
≥ 12 years	79	78.0	84.0
**Employment status**	-	-	-
Working	84	84.4	82.0
Not working	16	15.6	18.0
**Monthly income (ID)**	-	-	-
≤ 500,000	38	38.4	36.0
> 500,000	62	61.6	64.0
**Living place**	-	-	-
Rural	22	21.2	26.0
Urban	78	78.8	74.0
**Ever heard about osteoporosis**	-	-	-
No	7	7.2	6.0
Yes	93	92.8	94.0
**Osteoporosis diagnosis or screening ^c^**	-	-	-
No	82	80.8	88.0
Yes	18	19.2	12.0
self-reported osteoporosis ^b^ **	-	-	-
No	89.3	87.2	0.0
Yes	10.7	12.8	100.0
**Family history of osteoporosis ^c^**	-	-	-
No	72.7	72.4	74.0
Yes	27.3	27.6	26.0
**Family history of fracture**	-	-	-
No	59	58.0	64.0
Yes	41	42.0	36.0
**Smoking habit**	-	-	-
Not smoking	79.3	79.2	80.0
Smoking	20.7	20.8	20.0
**Alcohol habit**	-	-	-
Non alcoholic	99.3	99.6	98.0
alcoholic	0.7	0.4	2.0

IQD: Iraqi dinar; significant association between groups *P<0.05, **P<0.01; significant difference ^a^ *P*<0.001, ^b^ *P*<0.01, ^c^ *P*<0.05.

**Table 2:** Component matrix of exploratory factor analysis for osteoporosis self-efficacy scale Arabic version (OSES-A).

**Item**	**Pattern Matrix**		**Structure Matrix**		**Communalities**
-	**Factor 1**	**Factor 2**	**Factor 1**	**Factor 2**	
Question 1	**0.894**	-	**0.910**	-	0.829
Question 2	**0.950**	-	**0.924**	-	0.858
Question 3	**0.943**	-	**0.945**	-	0.894
Question 4	**0.932**	-	**0.923**	-	0.852
Question 5	**0.923**	-	**0.935**	-	0.876
Question 6	**0.891**	-	**0.907**	-	0.824
Question 7	-	**0.815**	-	**0.829**	0.689
Question 8	-	**0.883**	-	**0.877**	0.770
Question 9	-	**0.94**	-	**0.900**	0.821
Question 10	-	**0.915**	-	**0.907**	0.823
Question 11	-	**0.884**	-	**0.889**	0.790
Question 12	-	**0.760**	-	**0.812**	0.678
**Eigenvalues**	6.661	3.042	-	-	-
**% of variance**	55.51	25.35	-	-	**Total= 80.86%**
**Cronbach’s (α)**	0.966	0.935	-	-	**Total OSES-A= 0.927**

Extraction Method: Principal Axis Factoring, Rotation Method: Oblimin with Kaiser Normalization, Factor 1= OSES-Exercise-, Factor 2= OSES-Calcium, Items comprising each factor are in bold.

**Table 3:** Reliability test of the osteoporosis self-efficacy scale Arabic version (OSES-A).

**OSES-A Question No.**	**Mean**	**Standard Deviation**	**Corrected Item-Total Correlation**	**Cronbach's Alpha if Item Deleted**
Question 1	50.70	27.60	0.772	0.917
Question 2	47.93	27.96	0.725	0.919
Question 3	53.07	27.53	0.790	0.916
Question 4	48.03	28.34	0.748	0.918
Question 5	52.00	27.30	0.795	0.916
Question 6	54.43	27.65	0.769	0.917
Question 7	56.73	23.06	0.593	0.924
Question 8	59.50	21.90	0.597	0.924
Question 9	60.47	21.04	0.556	0.925
Question 10	60.20	20.85	0.618	0.923
Question 11	60.10	21.49	0.624	0.923
Question 12	55.27	21.35	0.648	0.922

Cronbach’s alpha was 0.927 for the total scale

**Table 4:** Multivariate regression analysis summary.

Variables Included		95% CI for Odds Ratio		
	B (SE)	Lower	Odds ratio	Upper
Constant	-0.879 (0.634)			
OKT-A (categorical)	1.219 (0.342) ^b^	1.730	3.384	6.619
OHBS-A (categorical)	0.916 (0.342) ^b^	1.279	2.499	4.882
Age (continuous)	-0.032 (0.014)^a^	0.942	0.969	0.997
Gender	-0.833 (0.342) ^a^	0.222	0.435	0.850

*Note*: *R*^2^ = 0.442 (Hosmer & Lemeshow), 0.118 (Cox & Snell), 0.198 (Nagelkerke). Model *_X_^2^*(4) = 37.52, *P*< 0.001. ^a^ *P*<0.05, ^**^ *P*<0.01.
